# Dynamic Virus-Dependent Subnuclear Localization of the Capsid Protein from a Geminivirus

**DOI:** 10.3389/fpls.2017.02165

**Published:** 2017-12-22

**Authors:** Liping Wang, Huang Tan, Mengshi Wu, Tamara Jimenez-Gongora, Li Tan, Rosa Lozano-Duran

**Affiliations:** ^1^Shanghai Center for Plant Stress Biology and Center for Excellence in Molecular Plant Science, Chinese Academy of Sciences, Shanghai, China; ^2^University of the Chinese Academy of Sciences, Beijing, China

**Keywords:** virus, TYLCV, nucleus, CP, nuclear speckles, nuclear foci, encapsidation, protein–protein interactions

## Abstract

Viruses are intracellular parasites with a nucleic acid genome and a proteinaceous capsid. Viral capsids are formed of at least one virus-encoded capsid protein (CP), which is often multifunctional, playing additional non-structural roles during the infection cycle. In animal viruses, there are examples of differential localization of CPs associated to the progression of the infection and/or enabled by other viral proteins; these changes in the distribution of CPs may ultimately regulate the involvement of these proteins in different viral functions. In this work, we analyze the subcellular localization of a GFP- or RFP-fused CP from the plant virus *Tomato yellow leaf curl virus* (TYLCV; Fam. *Geminiviridae*) in the presence or absence of the virus upon transient expression in the host plants *Nicotiana benthamiana* and tomato. Our findings show that, in agreement with previous reports, when the CP is expressed alone it localizes mainly in the nucleolus and weakly in the nucleoplasm. Interestingly, the presence of the virus causes the sequential re-localization of the CP outside of the nucleolus and into discrete nuclear foci and, eventually, into an uneven distribution in the nucleoplasm. Expression of the viral replication-associated protein, Rep, is sufficient to exclude the CP from the nucleolus, but the localization of the CP in the characteristic patterns induced by the virus cannot be recapitulated by co-expression with any individual viral protein. Our results demonstrate that the subcellular distribution of the CP is a dynamic process, temporally regulated throughout the progression of the infection. The regulation of the localization of the CP is determined by the presence of other viral components or changes in the cellular environment induced by the virus, and is likely to contribute to the multifunctionality of this protein. Bearing in mind these observations, we suggest that viral proteins should be studied in the context of the infection and considering the temporal dimension in order to comprehensively understand their roles and effects in the interaction between virus and host.

## Introduction

Viruses are intracellular parasites which, in their simplest form, comprise a nucleic acid genome and a proteinaceous capsid. Encapsidation (i.e., the enclosure of the viral genome within the viral capsid) protects the viral genome and is essential for viral transmission in nature. Viral capsids are formed through protein–protein and protein–genome interactions involving at least one virus-encoded capsid protein (CP). However, possibly as a requirement derived from the limited coding capacity of viruses, the CP is often multifunctional, as the rest of the viral proteins, and may play non-structural roles in many steps of the infection process. Multiple studies in the past decades have shown that the CP of plant viruses can act in processes such as delivery of the virus into the host cell, nuclear shuttling of viral genomes, viral replication, translation of viral proteins, viral movement within the host plant, or manipulation of host defenses, besides its canonical role in capsid formation and virus transmission [reviewed in (Bol, 2008; Callaway et al., 2001)]. The implication of the CP in one process or another might switch throughout the viral infection cycle, regulated perhaps by differential interactions with viral and host components. For example, viral replication and encapsidation have been proposed to be tightly linked physically.

Geminiviruses are insect-transmitted plant viruses with circular single-stranded (ss) DNA genomes, which are replicated in the nucleus of the host cell. Geminiviruses infect a wide range of plant species worldwide, including cash and staple crops, and currently pose a serious threat to food security. Despite the prevalence of geminiviruses in tropical and subtropical regions of the globe, the molecular mechanisms underlying pathogenicity by this family of viruses remain elusive.

In geminiviruses, a single CP forms the characteristic twin quasicosahedric viral capsid (Hatta and Francki, 1979; Zhang et al., 2001; Bottcher et al., 2004; Hipp et al., 2017). Besides its role in encapsidation, geminiviral CP is essential for transmission and determines vector specificity (Briddon et al., 1989, 1990; Noris et al., 1998; Liu et al., 1999; Hohnle et al., 2001). Moreover, CP can cooperatively bind ssDNA and double-stranded (ds) DNA in a sequence non-specific manner (Ingham et al., 1995; Liu et al., 1997; Palanichelvam et al., 1998; Hehnle et al., 2004; Priyadarshini and Savithri, 2009). Intriguingly, CP from different geminiviruses accumulates strongly in the nucleolus, and weakly in the nucleoplasm, when expressed in plant cells (Rojas et al., 2001; Unseld et al., 2001; Guerra-Peraza et al., 2005; Sharma and Ikegami, 2010). These experiments, however, have studied the localization of the CP in isolation, outside of the context of the infection. In animal viruses, there are examples of differential localization of CPs associated to the progression of the infection cycle or, more specifically, to the presence of other viral proteins. In the ssDNA Adeno-associated virus type 2 (AAV-2), capsid proteins are redistributed in nuclear bodies throughout the infection: five different stages, which may partially co-exist, can be distinguished (Wistuba et al., 1997). Capsids are pre-formed in the nucleolus, and later move to the nucleoplasm for virus encapsidation; the replication-associated protein, Rep, influences the nuclear distribution of the capsids (Wistuba et al., 1997). In the dsDNA human polyomavirus JC, one of the CPs, named VP1, is efficiently transported to the nucleus and localized in discrete nuclear speckles only in the presence of the other two CPs, VP2 and VP3 (Shishido-Hara et al., 2000). A similar case is that of Epstein-Barr virus, in which one of four CPs, BORF1, modifies the subcellular localization of the other three (Wang et al., 2015). These drastic virus-regulated changes in the subcellular distribution of CPs could at least partially underlie multifunctionality of this protein in a timely manner along the infection of a given cell. Whether the localization of the multifunctional geminivirus CP is also a dynamic process coordinated by the virus, as observed in the examples above, remains to be determined.

In this work, we analyze the subcellular localization of the CP from the geminivirus *Tomato yellow leaf curl virus* (TYLCV) (gen. *Begomoviridae*) upon transient expression of GFP-fused versions in its host plants *Nicotiana benthamiana* and tomato. Our findings show that, in agreement with previous reports, when the CP is not in the context of the viral infection but expressed alone, it localizes mainly in the nucleolus and weakly in the nucleoplasm. Strikingly, we have found that the presence of the virus causes the re-localization of the CP outside of the nucleolus and into discrete nuclear foci in a distinct sequence of stages, which consequently affects the sites of CP–CP homotypic interactions. In these foci, CP co-localizes with plant proteins involved in RNA metabolism. Expression of the viral replication-associated protein, Rep, is sufficient to exclude the CP from the nucleolus, but the localization of the CP in the characteristic patterns induced by TYLCV requires more than a single viral protein. Our results demonstrate that the subcellular distribution of viral proteins is a dynamic process, temporally regulated throughout the progression of the infection. This step-dependent differential regulation of the subcellular localization of viral proteins, which is determined by the presence of other viral components or changes in the cellular environment induced by them, is likely to contribute to multifunctionality.

## Materials and Methods

### Plasmids and Cloning

The Rep, C2, C3, C4, and V2 genes from TYLCV (GenBank accession number AJ489258) were cloned in pENTR-D/TOPO (Invitrogen) with stop codon and then Gateway-cloned into the binary vector pGWB2 (Wang et al., 2017). CP from TYLCV was cloned in pENTR-D/TOPO with and without stop codon and then Gateway-cloned into pGWB2, pGWB5 (C-terminal GFP fusion), pGWB6 (N-terminal GFP fusion), pGWB554 (C-terminal RFP fusion) and pGWB555 (N-terminal RFP fusion) (Nakagawa et al., 2007a,b; Wang et al., 2017). Primers and plasmids used in this work are listed in **Supplementary Table S1**. A partial TYLCV dimer (1.2 genomes) comprising two intergenic regions was cloned in pENTR-D/TOPO and Gateway-cloned into the binary vector pGWB501 to generate the infectious clone (Rosas-Diaz et al., unpublished).

The plasmids CP-YFPn and CP-YFPc used for biomolecular fluorescent complementation (BiFC) are generated by Gateway cloning from TOPO-CP-NS into pGTQL1211YN and pGTQL1221YC (Lu et al., 2010).

Nuclear markers used in this project belong to The Plant Nuclear Marker Collection of NASC^1^.

### Plant Material

*Nicotiana benthamiana* and tomato (cv. Moneymaker) plants were grown in a controlled growth chamber in long day conditions (16 h light/8 h dark) at 25°C.

### Agrobacterium-Mediated Transient Transformation

The TYLCV infectious clone or gene expression vectors were transformed into *Agrobacterium tumefaciens* strain GV3101. Agrobacterium cells carrying these constructs were liquid cultured in LB with appropriate antibiotics at 28°C overnight. Bacterial cultures were centrifuged at 4,000 *g* for 10 min and resuspended in the infiltration buffer (10 mM MgCl_2_, 10 mM MES pH 5.6, 150 μM acetosyringone) to an OD_600_ = 0.5–1. Bacterial suspensions were incubated in the buffer at room temperature and in the dark for 4 h before using them to infiltrate 4-week-old *N. benthamiana* and 4-week-old tomato plants. For co-infiltration experiments, the *Agrobacterium* suspensions carrying different constructs were mixed at 1:1 ratio before infiltration.

### Confocal Microscopy

Confocal imaging of CP-GFP, GFP-CP, CP-RFP, and RFP-CP in *N. benthamiana* epidermal cells was performed on a Leica TCS SP8 point scanning confocal microscope using the pre-set settings for GFP (with Ex:488 nm, Em:500–550 nm) or for RFP (with Ex:561 nm, Em:600–650 nm).

Confocal imaging of RFP-CP in tomato epidermal cells was performed on a Leica TCS SP8 point scanning confocal microscope using the pre-set settings for RFP with Ex:554 nm, Em:580–630 nm, HyD gating from 1–10 ns on TCS SP8 SMD FLCS.

Confocal imaging for co-localization of CP-GFP or CP-RFP with nuclear markers in *N. benthamiana* epidermal cells was performed on a Leica TCS SP8 point scanning confocal microscope using the pre-set sequential scan settings for GFP with Ex:488 nm, Em:500–550 nm and for RFP with Ex:561 nm, Em:600–650 nm.

### Quantitative PCR (qPCR) and Reverse Transcription PCR (qRT-PCR)

RNA was extracted from five 8 mm leaf disks using the Plant RNA kit (OMEGA Bio-tek # R6827); cDNA was prepared using the iScript^TM^ cDNA Synthesis Kit (Bio-Rad #1708890) according to the manufacturer’s instructions.

DNA was extracted with 2xCTAB from leaf tissues at different times after infiltration. Quantitative PCR to determine viral accumulation was performed with primers to amplify Rep (**Supplementary Table S2**).

DNA and cDNA were analyzed by qPCR with iTaq^TM^ Universal SYBR^®^ Green Supermix (Bio-Rad, #1725120). The reactions were done as follows: 3 min at 95°C, 40 cycles consisting of 15 s at 95°C, 30 s at 60°C. The primers used to amplify the viral genes are described in **Supplementary Table S2**. As an internal reference for DNA and RNA detection, the 25S ribosomal DNA interspacer (ITS) was used (Mason et al., 2008).

### Time Course Assay

**FIGURE 1:**
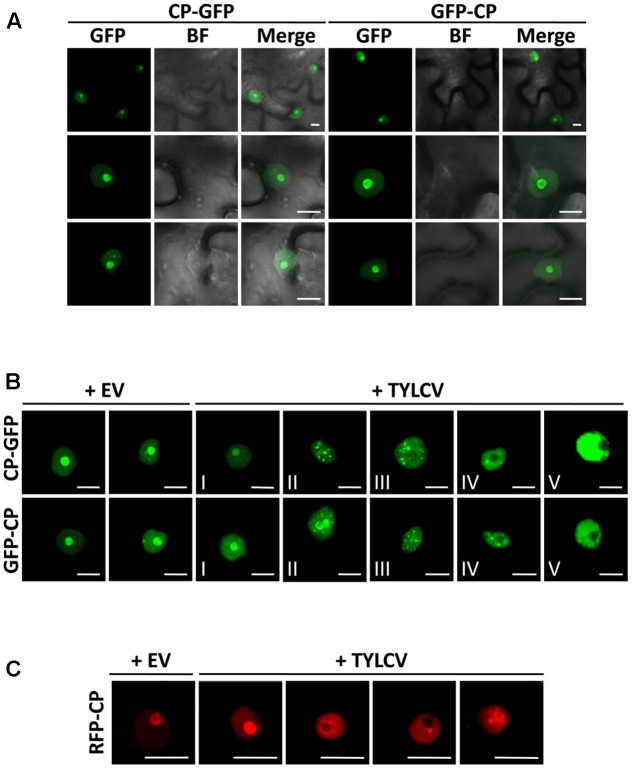
The capsid protein (CP) from *Tomato yellow leaf curl virus* (TYLCV) localizes to the nucleolus and the nucleoplasm, and this localization is changed in the presence of the virus. *Nicotiana benthamiana* leaves were infiltrated with *Agrobacterium tumefaciens* carrying constructs to express CP-GFP and GFP-CP alone **(A)** or co-infiltrated with *A. tumefaciens* carrying a TYLCV infectious clone or an empty vector (EV) as control **(B)**. The subcellular localization of CP-GFP or GFP-CP was observed under the confocal microscope 2 days after infiltration. Five subcellular localization stages of GFP-fused CP were defined. Stage I: GFP-fused CP localizes strongly to the nucleolus and more weakly to the nucleoplasm, and only occasionally forms a few speckles in a few cells (<5%); Stage II: GFP-fused CP shows a similar localization similar to that of Stage I, but forms numerous speckles in the nucleoplasm; Stage III: GFP-fused CP shows a localization similar to that of Stage II, but it is absent from the nucleolus; Stage IV: GFP-fused CP localizes to the nucleoplasm only, where it is unevenly distributed; Stage V: GFP-fused CP is uniformly distributed in the nucleoplasm. This experiment was done three times; more than 20 cells were observed per sample and replicate. **(C)** Tomato leaves were co-infiltrated with *A. tumefaciens* carrying a construct to express RFP-CP, and a TYLCV infectious clone or empty vector (EV) as control. The subcellular localization of RFP-CP was observed under the confocal microscope 2 days after infiltration. This experiment was repeated three times; more than 15 cells were observed per sample and replicate. BF, Bright field. Scale bar: 10 μm.

Three *N. benthamiana* plants were co-infiltrated with *A. tumefaciens* clones containing constructs to express CP-GFP, and clones carrying a TYLCV infectious clone or an empty vector (EV). At different time points between 20 and 48 h post-infiltration, CP-GFP-expressing cells were imaged under the confocal microscope and the number of cells in different stages according to the CP localization (see **Figure 1B**) was determined. At each time point, 3 to 5 leaf disks from at least two independent plants were observed and more than 90 cells were imaged. After observation, the leaf disks were collected to determine viral accumulation.

## Results

### The CP from TYLCV Changes Its Subnuclear Localization in the Presence of the Virus

Using transient expression of CP-GFP and GFP-CP fusion proteins in *N. benthamiana* we could observe that, as previously described, CP from TYLCV localizes to the nucleolus, and weakly to the nucleoplasm (Rojas et al., 2001; **Figure 1A**); a similar subcellular distribution has also been shown for other geminiviral CPs (Unseld et al., 2001; Guerra-Peraza et al., 2005; Sharma and Ikegami, 2010). Accumulation of the CP in one or few speckles in the nucleoplasm can occasionally be observed (**Figures 1A,B**). In order to determine whether the viral infection may change the subcellular localization of the CP, we co-infiltrated a TYLCV infectious clone together with the clones to express CP-GFP or GFP-CP. Infiltration of this infectious clone results in effective viral replication in the agrobacterium-transformed cells, therefore mimicking the cellular environment during a natural viral infection. Strikingly, we found that the presence of the virus drastically modifies the distribution of the CP in the nucleus: in infected cells, the CP is excluded from the nucleolus and can be detected in numerous strong nuclear speckles or becomes unevenly distributed in the nucleoplasm (**Figure 1B**). According to the distribution of CP in the nucleus, we could distinguish five different stages (**Figure 1B**): stage I resembles the localization of the CP when expressed alone, which accumulates mostly in the nucleolus; in stage II, the CP can still be detected in the nucleolus, but forms multiple intense speckles in the nucleoplasm; in stage III, speckles can still be detected, but the CP is absent from the nucleolus; in stage IV, speckles become blurred, and the nucleoplasmic signal becomes stronger but unevenly distributed; in stage V, discrete speckles can no longer be detected, and the nucleoplasm shown a strong and more evenly distributed signal. This effect of the presence of the virus on the localization of the CP can also be detected in *N. benthamiana* and tomato upon transient expression of a RFP-CP fusion protein (**Figure 1C** and **Supplementary Figure S1**).

**FIGURE 2:**
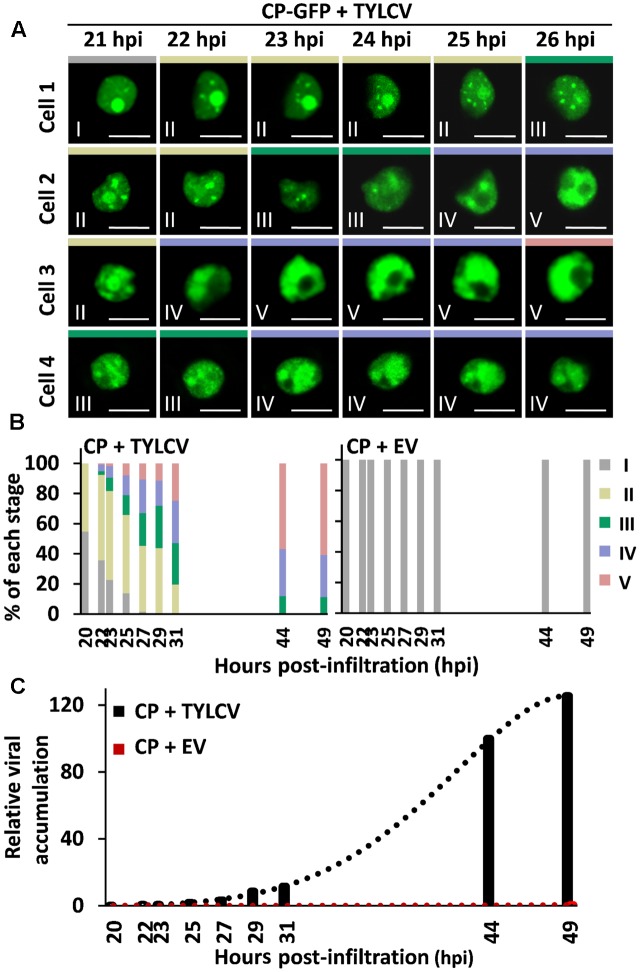
Succession of stages of CP localization in the presence of the virus. *N. benthamiana* leaves were co-infiltrated with *A. tumefaciens* carrying a construct to express CP-GFP, and a TYLCV infectious clone or an empty vector (EV) as control. **(A)** The subcellular localization of CP-GFP in four independent cells was observed under the confocal microscope every 15 min from 21 h post-infiltration (hpi) to 27 hpi; representative images are shown. Scale bar: 10 μm. The settings used for imaging each of the cells were unchanged for the duration of the time course. **(B)** Percentage of each stage of subcellular localization of CP-GFP in *N. benthamiana* leaves at different time points, as indicated. For each time point, *n* > 90 cells. This experiment was repeated three times with similar results. **(C)** TYLCV DNA accumulation at different time points, as indicated. For each time point, samples were collected after observation under the confocal microscope in **(B)**. Viral DNA was extracted and quantified by quantitative PCR (qPCR) with primers to amplify the Rep gene. The 25S ribosomal DNA interspacer (ITS) was used as normalizer. The amount of viral DNA is represented relative to ITS. This experiment was repeated twice with similar results.

Time-course experiments allowed us to establish the temporal sequence of the stages of CP localization: as shown in **Figure 2**, CP-GFP progresses through stages I–II–III–IV–V in individual cells (**Figure 2A**), and different stages coexist in the cell population (**Figure 2B**); the overall progression of stages correlates with viral DNA accumulation (**Figure 2C**), suggesting that it occurs as part of or in parallel to the development of the infection.

### The Presence of the Virus Affects the Site of CP–CP Interactions

**FIGURE 3:**
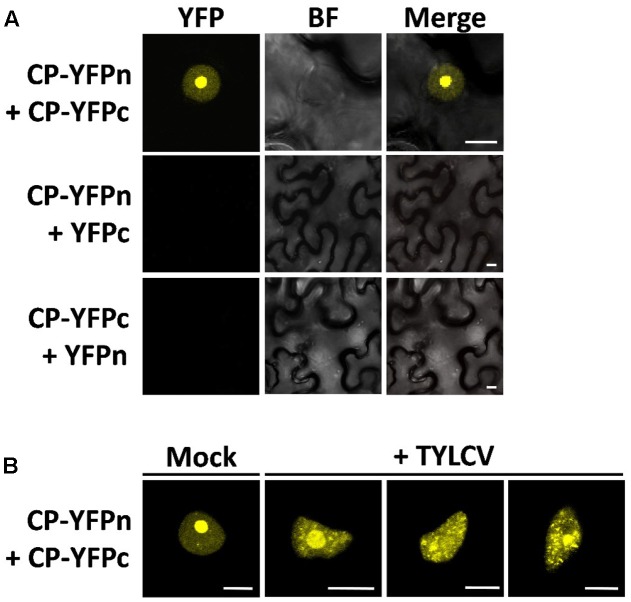
The presence of the virus affects the sites of CP–CP homotypic interactions. CP–CP homotypic interactions detected by bimolecular fluorescent complementation (BiFC) assay. *N. benthamiana* leaves were co-infiltrated with *A. tumefaciens* carrying constructs to express CP-YFPn (CP fused to the N-terminal half of the YFP) and CP-YFPc (CP fused to the C-terminal part of the YFP) **(A)**. In **(B)**, CP-YFPn and CP-YFPc were co-expressed with a TYLCV infectious clone or mock control. Samples were observed under the confocal microscope 2 days after infiltration; CP–CP interactions are detected as yellow fluorescence. This experiment was repeated three times; more than 15 cells were observed per sample and replicate. BF, Bright field. Scale bar: 10 μm.

The CP from geminiviruses has been shown to interact with itself (CP–CP or homotypic interaction) (Hallan and Gafni, 2001), which may be relevant for assembly of the viral capsid and perhaps other roles of the CP. In order to determine where in the cell this homotypic interaction is taking place, and whether it changes localization in the presence of the virus, following the changes in CP distribution, we decided to use bimolecular fluorescence complementation (BiFC). As shown in **Figure 3**, in the absence of the virus the CP–CP interaction can be detected as YFP signal strongly in the nucleolus and weakly in the nucleoplasm, mirroring the distribution of the CP. In the presence of the virus, however, the sites of CP–CP interaction shift, weakening in or disappearing from the nucleolus, and appearing as distinct speckles in the nucleoplasm (**Figure 3B**), which correlates with the observed localization of CP in TYLCV-infected cells (**Figure 1B**).

### The Changes in CP Localization Induced by TYLCV Do Not Depend on a Single Viral Protein, But Rep Is Sufficient to Exclude CP from the Nucleolus

**FIGURE 4:**
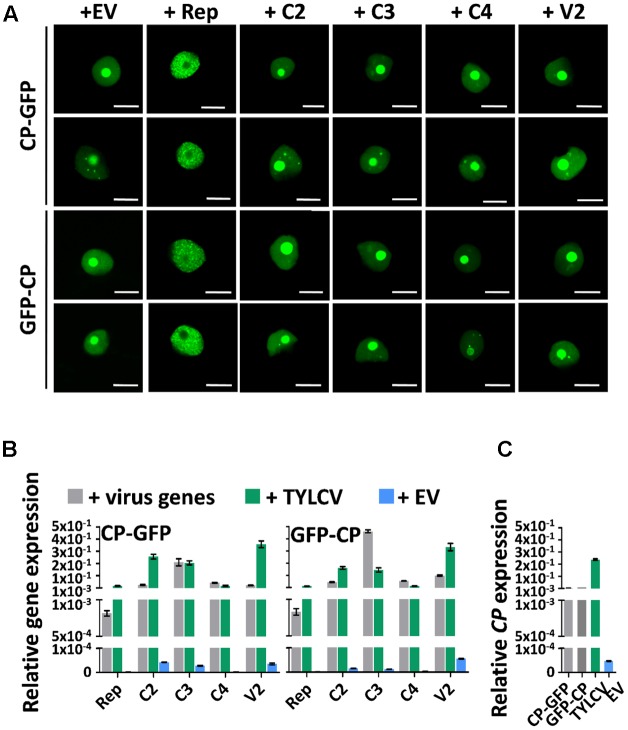
The changes in CP localization induced by TYLCV do not depend on a single viral protein. *N. benthamiana* leaves were co-infiltrated with *A. tumefaciens* carrying constructs to express CP-GFP or GFP-CP, and constructs to express each other virus protein independently (Rep, C2, C3, C4, and V2) or empty vector (EV) as control. **(A)** The subcellular localization of CP-GFP (upper panels) or GFP-CP (lower panels) was observed under the confocal microscope 2 days after infiltration. This experiment was repeated three times with similar results; more than 20 cells were observed per sample and replicate. Scale bar: 10 μm. **(B)** Expression of viral genes in the samples in **(A)**, measured by quantitative reverse transcription PCR (qRT-PCR) 2 days after infiltration. The samples were collected after observation under the confocal microscope. The 25S ribosomal DNA interspacer (ITS) was used as normalizer. The expression of viral genes is represented relative to ITS. Gray columns show the virus gene expression level when each viral gene is independently co-expressed with CP-GFP (left) or GFP-CP (right) from a binary vector. Blue columns show the virus gene expression level when a TYLCV infectious clone is co-infiltrated with constructs to express CP-GFP (left) or GFP-CP (right). Green columns represent the EV control. **(C)** Expression of the CP gene in *N. benthamiana* leaves transiently expressing CP-GFP, GFP-CP, TYLCV, or empty vector, measured by qRT-PCR 2 days after infiltration. The expression of the CP gene is presented relative to normalizer ITS. The average values (±standard deviation) from three technical repeats of qRT-PCR are shown. This experiment was repeated three times with similar results.

Since the presence of TYLCV results in changes in the subnuclear localization of the CP, and the virus encodes another five proteins, we wondered whether, as described for Adeno-associated virus type 2, human polyomavirus JC, or Epstein-Barr virus, another of the viral proteins enables the re-localization of the CP. In order to determine whether this is the case, we transiently expressed CP-GFP or GFP-CP in *N. benthamiana* leaves together with each of the viral proteins independently, or with a mock control. As shown in **Figure 4A**, none of the viral proteins was capable of recapitulating the TYLCV-induced changes in the localization of the CP. Nevertheless, the Rep protein was sufficient to exclude the CP from the nucleolus, leading to a strong, uneven distribution of the CP in the nucleoplasm (**Figure 4A**). Expression of the viral genes from the binary vectors was confirmed by quantitative reverse transcription PCR (qRT-PCR), and found similar to the native expression during the viral infection or lower (**Figures 4B,C**). The inability of individual viral proteins to mimic the changes produced by the virus in the localization of the CP suggest that either a combination of more than one viral protein or the viral genome or a related process, such as viral DNA replication and/or encapsidation, are required for this effect.

### The Virus-Induced CP-Containing Nuclear Foci Partially Co-localize with Markers of Sites of RNA Processing

**FIGURE 5:**
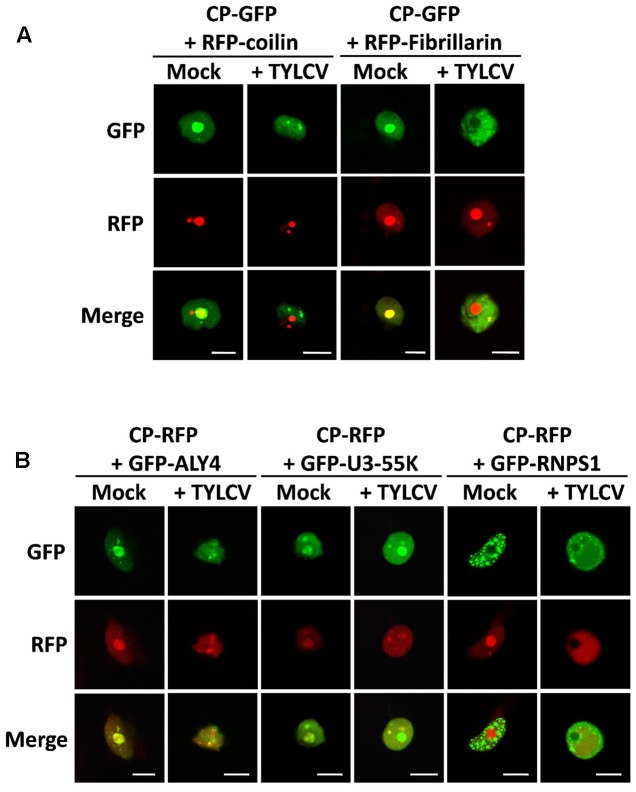
The CP-containing nuclear foci induced by TYLCV partially co-localize with markers of sites of RNA processing. *N. benthamiana* leaves were co-infiltrated with *A. tumefaciens* carrying constructs to express CP-GFP **(A)** or CP-RFP **(B)** together with marker proteins of subnuclear compartments: coilin and fibrillarin fused to RFP **(A)**, and ALY4, U3-55K, and RNPS1 fused to GFP **(B)**. (For details about these marker proteins, see the “Results” section). Samples were observed under the confocal microscope 2 days after infiltration using sequential scanning. This experiment was repeated three times; more than 20 cells were observed per sample and replicate. Scale bar: 10 μm.

Within the cell nucleus, distinct non-membrane-bound structures or bodies of different numbers and sizes, which may vary between cell types and depending on developmental or environmental conditions, can be distinguished. Although the most prominent of these structures is the nucleolus, a number of other subnuclear domains have been characterized based on their protein and nucleic acid composition, which determines their function; some examples are Cajal bodies, splicing speckles, or photo bodies. In order to investigate whether the CP-containing nuclear speckles formed upon TYLCV infection correspond to some of the known subnuclear structures, we decided to co-express the GFP- or RFP-fused CP, with or without TYLCV, with established nuclear markers that are found distributed in nucleoplasmic bodies. For this purpose, we selected coilin, fibrillarin, ALWAYS EARLY 4 (ALY4), RNPS1 (also known as SR45), and U3-55K (also known as YAOZHE) as marker proteins. Coilin localizes to the nucleolus and Cajal bodies (Collier et al., 2006), similar to fibrillarin (Barneche et al., 2000) (**Figure 5**). ALY4 labels the nucleolus and discrete nuclear speckles (Pendle et al., 2005; **Figure 5**), and, in mammals and possibly also in plants, is a component of the exon junction complex (EJC), which plays a central role in mRNA biogenesis (Boehm and Gehring, 2016). Also part of the EJC is RNPS1, which localizes in distinct foci in the nucleoplasm (**Figure 5**). U3-55K labels the nucleolus and nucleoplasmic bodies (Pendle et al., 2005; **Figure 5**), and, in yeast and human, is a component of the U3 small nucleolar ribonucleoprotein (snoRNP) complex, which functions in 18S rRNA processing (Watkins and Bohnsack, 2012); U3-55K has been proposed to have a similar role in plants (Li et al., 2010, BMC Plant Biology). As shown in **Figure 5**, the CP co-localizes with coilin, fibrillarin, ALY4, and U3-55K in the nucleolus when expressed alone; however, in the presence of the virus, the CP partially co-localizes in nucleoplasmic speckles with ALY4 and U3-55K only. Given the assumed roles of ALY4 and U3-55K in mRNA and rRNA biogenesis and processing, respectively, these results suggest the possibility that the CP may interfere with RNA metabolism in the infected cells.

Curiously, we found that the presence of the virus, but not the CP alone, seems to alter the localization of ALY4, since this protein can no longer be detected in the nucleolus (**Figure 5** and **Supplementary Figure S2**). Although an effect of the virus on the subnuclear distribution of GFP-RNPS1 cannot be ruled out, variability on the localization pattern of this marker protein makes it difficult to determine whether this is indeed the case (**Supplementary Figure S3**).

## Discussion

Viral proteins are generally multifunctional, and their targeting to different subcellular compartments may at least partially underpin their different functions. A recent example of this localization-dependent functional specification is that of the C4 protein from TYLCV, which localizes to plasma membrane and chloroplasts; only plasma membrane-localized C4 can prevent the cell-to-cell spread of RNA interference, while chloroplastic C4 seems to modulate hormone-based defense responses (Rosas-Diaz et al., unpublished). Viral CPs are the building blocks of the viral capsid, but in addition to this structural role they can play a number of others throughout the viral infection. Examples in animal viruses exist demonstrating that CPs can re-localize within the cell in the presence of other viral proteins or during the viral infection (Wistuba et al., 1997; Shishido-Hara et al., 2000; Wang et al., 2015). In the case of the geminivirus TYLCV, subcellular localization of the CP has been previously studied (Rojas et al., 2001); however, in these experiments the CP was expressed in isolation, and therefore in the absence of other viral proteins, the viral genome, and the cellular changes triggered by the viral infection. In this work, we show that the localization of the CP is dynamic, changing in the presence of the virus in a temporal sequence in which several stages can be distinguished (**Figures 1, 2**). This opens a new perspective to the study of geminiviral proteins: our results indicate that the subcellular localization of geminiviral proteins is actually a dynamic, regulated process, and imply that viral proteins should be considered in the context of the infection and adding the temporal dimension in order to comprehensively study their roles and effects in the interaction between virus and host. It should be considered, nevertheless, that fusion proteins (GFP-CP, CP-GFP, RFP-CP, CP-RFP) are used in this work; although the results are similar for all tagged versions of the CP, and it is not immediately obvious how a tag would lead to the observed virus-induced changes in the subnuclear localization of this protein, the fact that we are not imaging the untagged CP should be kept in mind.

Interestingly, our results are reminiscent of those obtained by Wistuba et al. (1997) when studying the subnuclear localization of the capsid proteins from AAV-2, which show a redistribution from the nucleolus to nuclear bodies following the progression of the infection and an influence of Rep proteins in the distribution of viral capsids. Given that both AAV-2 and TYLCV have ssDNA genomes that have to be replicated and encapsidated in the nucleus, this virus- and Rep-dependent modulation of the localization, and hence potential functions, of the CP might represent a relevant part of the cycle of ssDNA viruses.

When expressed alone, CP from TYLCV, as previously described, localizes mainly to the nucleolus (**Figures 1, 3, 4**). How proteins are targeted to this subnuclear structure is poorly understood, although Nucleolar Localization Signals (NoLS) have been identified. Interestingly, one NoLS is predicted in the CP protein, between positions 37 and 62, according to the Nucleolar localization sequence Detector (Scott et al., 2011); in this case, the putative NoLS does not overlap with the defined Nuclear Localization Signal (Kunik et al., 1998). The biological significance of the targeting of the CP to the nucleolus is still unclear; notably, nucleolar abnormalities have been observed in TYLCV-infected cells (Kim et al., 1978; Cherif and Russo, 1983; Channarayappa et al., 1992).

In some cases, the CP can be observed in one or few nucleoplasmic bodies in the absence of the virus. Lack of co-localization with fibrillarin and coilin indicate that these CP-positive structures do not correspond to the Cajal body. The partial co-localization with U3-55K suggests that these speckles might be sites of pre-rRNA processing, raising the idea that the CP might interfere with the regulation of ribosome biogenesis.

We have observed that co-expression with Rep is sufficient to trigger nucleolar exclusion of the CP and its dotted distribution in the nucleoplasm (**Figure 4**); however, and since Rep is not sufficient to recapitulate all different stages of CP localization that are distinguishable upon TYLCV infection, it is possible that active viral replication/encapsidation is required for this. A protein–protein interaction between Rep and the CP has been described for the geminivirus *Mung bean yellow India virus* (Malik et al., 2005), which may serve to physically link replication and encapsidation of the viral genome to coordinately regulate these interdependent processes. The modification of the localization of a viral protein in the presence of another has been described before for CI and P3N-PIPO from the potyvirus *Turnip mosaic virus* (Wei et al., 2010).

During our co-localization experiments, we observed that the presence of TYLCV, but not the CP alone, altered the subnuclear localization of the possible EJC component ALY4: this protein, which is normally found in the nucleolus, is frequently excluded from this structure in TYLCV-infected cells (**Figure 5B** and **Supplementary Figure S2**). It is interesting to note that changes in the localization of ALY proteins, including ALY4, have been reported upon co-expression with Tomato bushy stunt virus p19 protein (Uhrig et al., 2004); in this case, ALY4 relocalizes from the nucleus to the cytoplasm. It would be interesting to determine whether exclusion of ALY4 from the nucleolus is a general virulence strategy of plant viruses. Recently, ALY4 has been shown to participate in the regulation of pathogen-induced hypersensitive response (HR) in *N. benthamiana* and Arabidopsis (Teng et al., 2014), and therefore assigned a role in plant-pathogen interactions.

## Author Contributions

LW, HT, MW, TJ-G, and LT performed the experiments. All authors contributed to experimental design and interpretation. RL-D conceived the project and wrote the manuscript with contributions from all authors.

## Conflict of Interest Statement

The authors declare that the research was conducted in the absence of any commercial or financial relationships that could be construed as a potential conflict of interest.

## Footnotes

1 http://arabidopsis.info/CollectionInfo?id=126

## Supplementary Material

FIGURE S1 Localization of CP-RFP and RFP-CP in the presence or absence of the virus in transiently transformed *Nicotiana benthamiana* leaves. *N. benthamiana* leaves were infiltrated with *Agrobacterium tumefaciens* carrying a construct to express CP-RFP/RFP-CP alone or co-infiltrated with *A. tumefaciens* carrying a TYLCV infectious clone. The subcellular localization of CP-RFP or RFP-CP was observed under the confocal microscope 2 days after infiltration. This experiment was repeated three times; more than 20 cells were observed per sample and replicate. Scale bar: 10 μm.

FIGURE S2 Subcellular localization of GFP-ALY4 in the presence or absence of TYLCV. *N. benthamiana* leaves were infiltrated with *A. tumefaciens* carrying a construct to express GFP-ALY4 alone **(A)** or co-infiltrated with *A. tumefaciens* carrying a TYLCV infectious clone **(B)**. The subcellular localization of GFP-ALY4 was observed under the confocal microscope 2 days after infiltration. This experiment was repeated three times; more than 20 cells were observed per sample and replicate. Scale bar: 10 μm.

FIGURE S3 Subcellular localization of GFP-RNPS1 in the presence or absence of TYLCV. *N. benthamiana* leaves were infiltrated with *A. tumefaciens* carrying a construct to express GFP-RNPS1 alone **(A)** or co-infiltrated with *A. tumefaciens* carrying a TYLCV infectious clone **(B)**. The subcellular localization of GFP-RNPS1 was observed under the confocal microscope 2 days after infiltration. This experiment was repeated three times; more than 20 cells were observed per sample and replicate. Scale bar: 10 μm.

TABLE S1 Plasmids and cloning primers list.

TABLE S2 Primers used for qPCR.
