# Supplementary figures and images for: Dynamic Virus-Dependent Subnuclear Localization of the Capsid Protein from a Geminivirus
Source: Front Plant Sci. 2017 Dec 22;8:2165. doi: 10.3389/fpls.2017.02165 (PMC5744400; doi:10.3389/fpls.2017.02165)

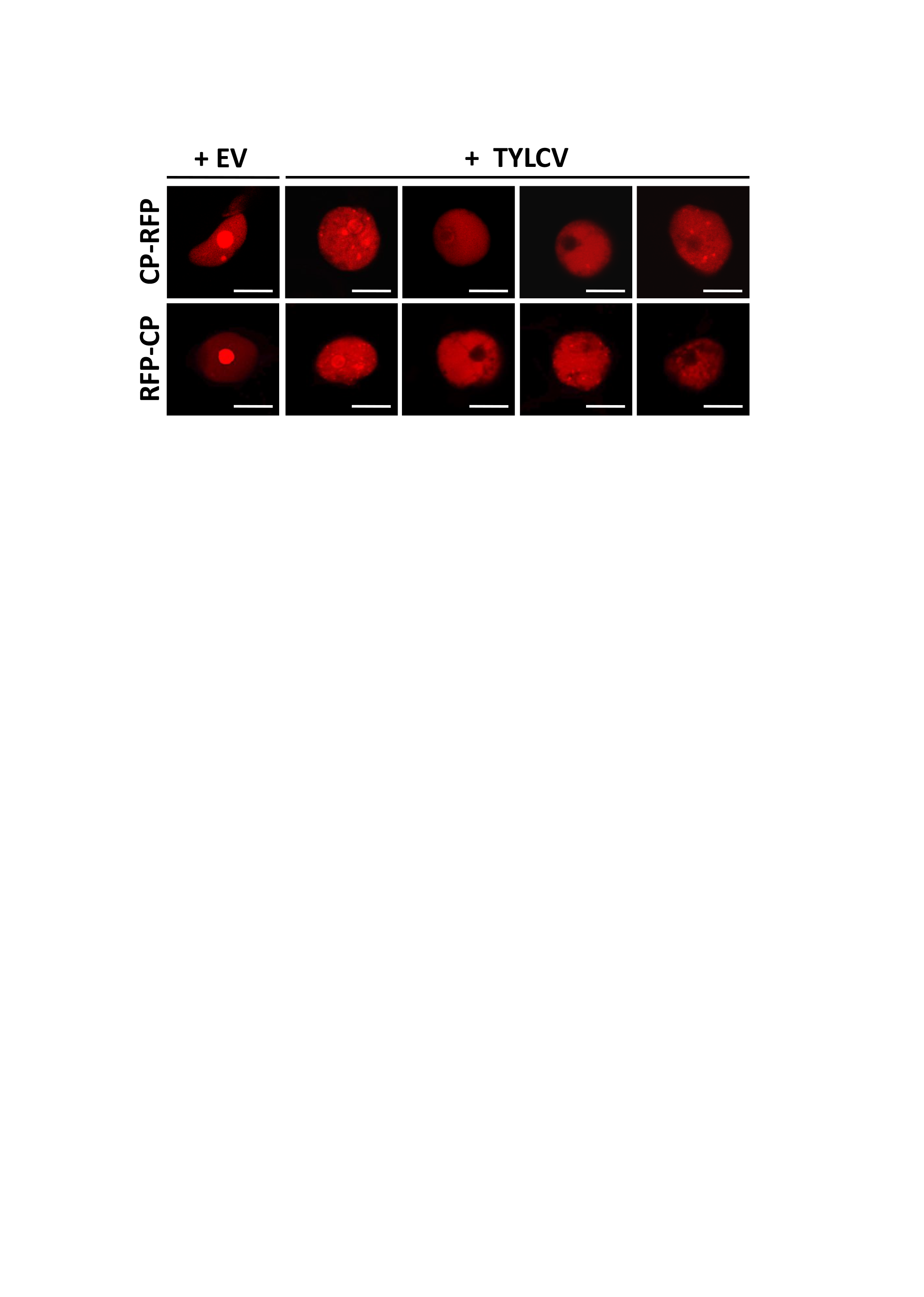

Supplement: FIGURE S1 — Localization of CP-RFP and RFP-CP in the presence or absence of the virus in transiently transformed Nicotiana benthamiana leaves. N. benthamiana leaves were infiltrated with Agrobacterium tumefaciens carrying a construct to express CP-RFP/RFP-CP alone or co-infiltrated with A. tumefaciens carrying a TYLCV infectious clone. The subcellular localization of CP-RFP or RFP-CP was observed under the confocal microscope 2 days after infiltration. This experiment was repeated three times; more than 20 cells were observed per sample and replicate. Scale bar: 10 μm. [file Image_1.TIF]

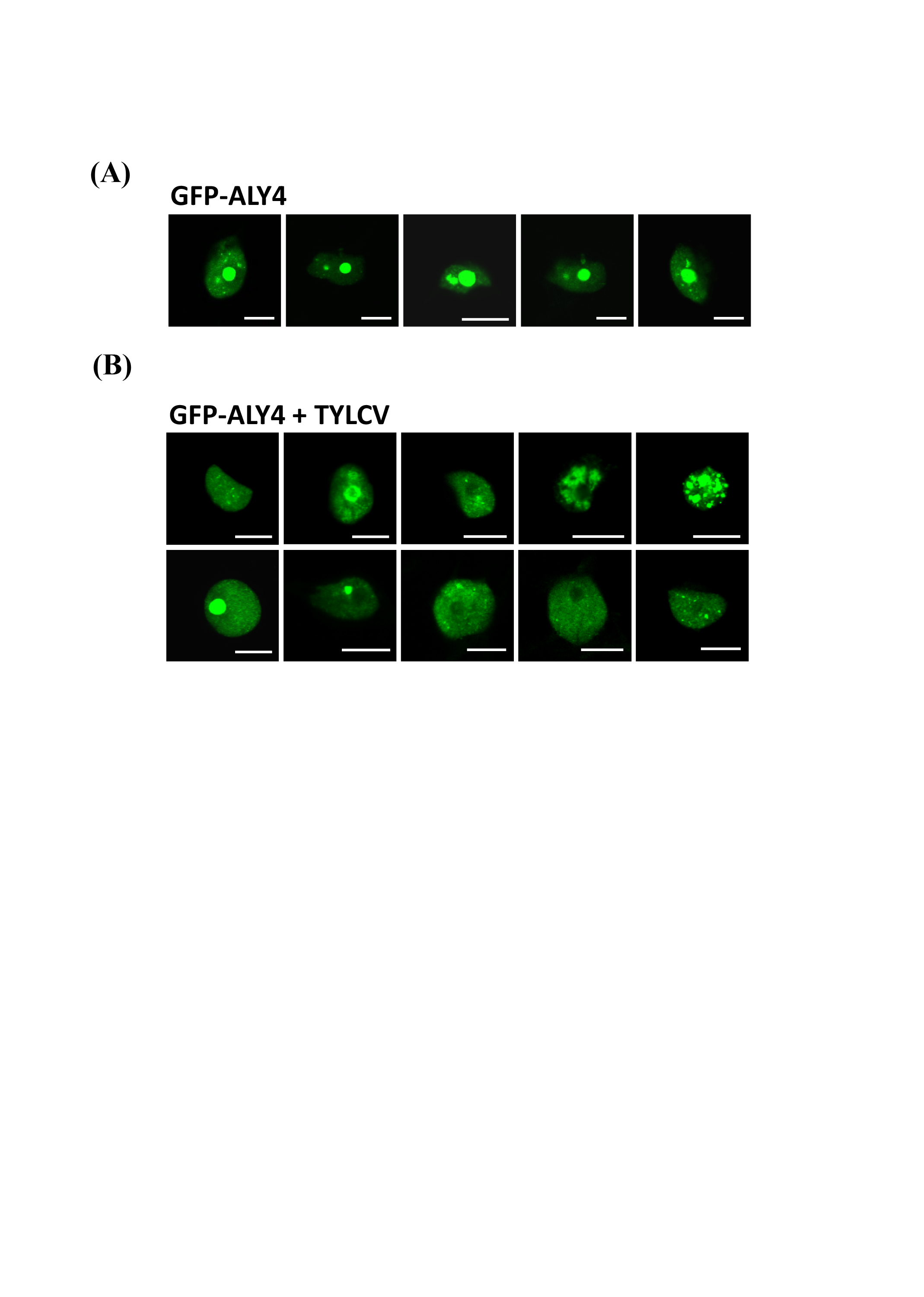

Supplement: FIGURE S2 — Subcellular localization of GFP-ALY4 in the presence or absence of TYLCV. N. benthamiana leaves were infiltrated with A. tumefaciens carrying a construct to express GFP-ALY4 alone (A) or co-infiltrated with A. tumefaciens carrying a TYLCV infectious clone (B). The subcellular localization of GFP-ALY4 was observed under the confocal microscope 2 days after infiltration. This experiment was repeated three times; more than 20 cells were observed per sample and replicate. Scale bar: 10 μm. [file Image_2.TIF]

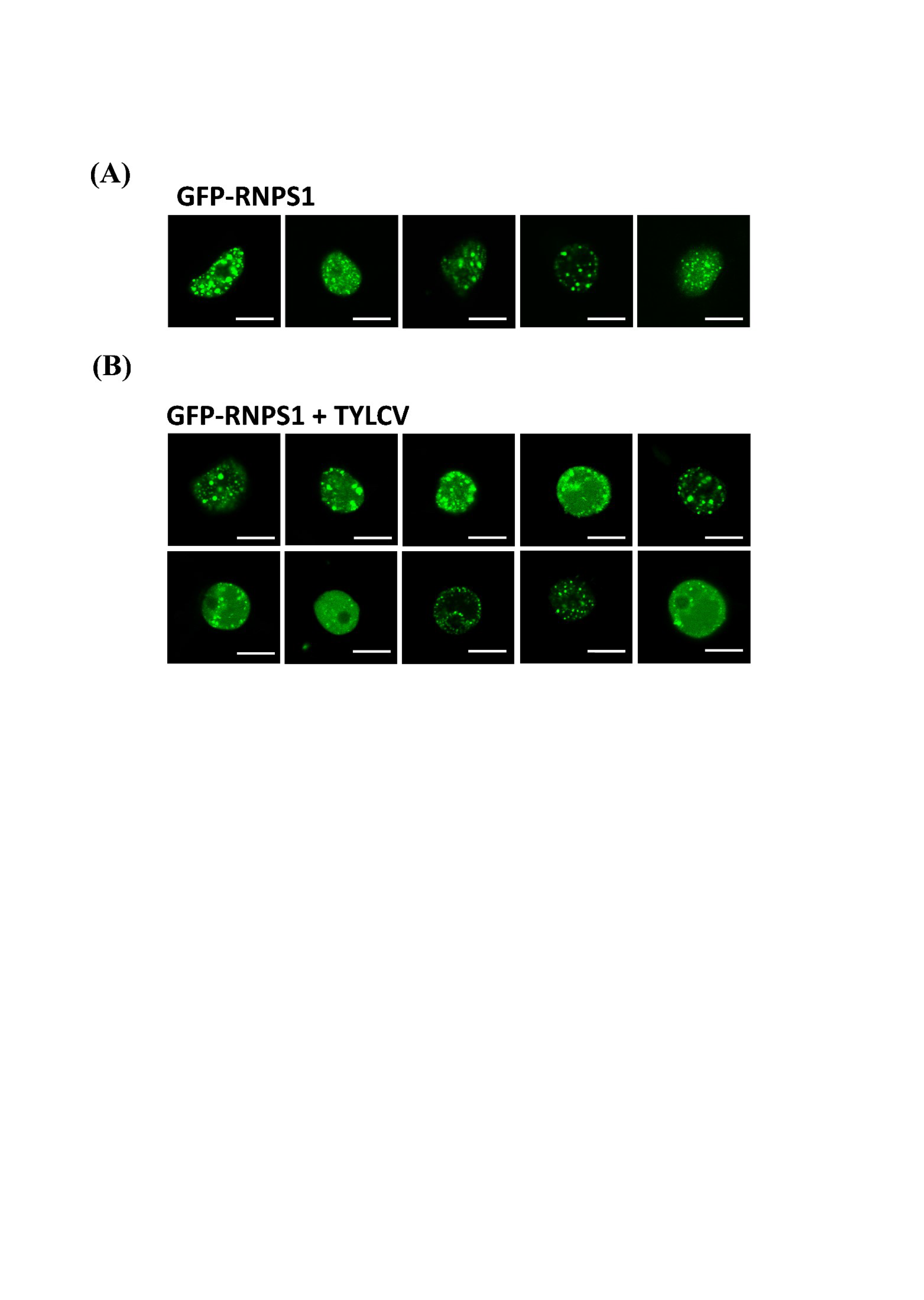

Supplement: FIGURE S3 — Subcellular localization of GFP-RNPS1 in the presence or absence of TYLCV. N. benthamiana leaves were infiltrated with A. tumefaciens carrying a construct to express GFP-RNPS1 alone (A) or co-infiltrated with A. tumefaciens carrying a TYLCV infectious clone (B). The subcellular localization of GFP-RNPS1 was observed under the confocal microscope 2 days after infiltration. This experiment was repeated three times; more than 20 cells were observed per sample and replicate. Scale bar: 10 μm. [file Image_3.TIF]
