# Supplementary material for: Dynamic Virus-Dependent Subnuclear Localization of the Capsid Protein from a Geminivirus
Source: Front Plant Sci. 2017 Dec 22;8:2165. doi: 10.3389/fpls.2017.02165 (PMC5744400; doi:10.3389/fpls.2017.02165)
Supplement: TABLE S1 — Plasmids and cloning primers list. [file Table_1.PDF]

Supplementary table 1: Plasmids and cloning primers list

Plasmid name	Vector Entry	Vector backbone	Primers to clone in entry vectors
TOPO-Rep (with stop codon)	pENTRD/TOPO (Invitrogen)		**F:** CACCATGCCTCGTTTATTTAA<br>**R:** TTACGCCTTATTGGTTTC
TOPO-C2 (with stop codon)	pENTRD/TOPO (Invitrogen)		**F:** CACCATGCAACCTTCGTC<br>**R:** CTAAATACTCTTAAG
TOPO-C3(with stop codon)	pENTRD/TOPO (Invitrogen)		**F:** CACCATGGATTCACGCACAG<br>**R:** TTAATAAAATTTATATT
TOPO-C4 (with stop codon)	pENTRD/TOPO (Invitrogen)		**F:** CACCATGGGAACCACATC<br>**R:** TTAATATATTGAGGG
TOPO-V2 (with stop codon)	pENTRD/TOPO (Invitrogen)		**F:** CACCATGTGGGACCCACTTC<br>**R:** TCAGGGCTTCGATAC
TOPO-CP(with stop codon)	pENTRD/TOPO (Invitrogen)		**F:** CACCATGTCGAAGCGACCAG<br>**R:** TTAATTTGATATTGAATC
TOPO-CP(without stop codon)	pENTRD/TOPO (Invitrogen)		**F:** CACCATGTCGAAGCGACCAG<br>**R:** ATTTGATATTGAATC
TOPO-TYLCV			1.2 copies of TYLCV genome. Full viral genome with another IR (2334 - 2781 nt plus 1 -189 nt)
35S:Rep	TOPO-Rep-S	pGWB2	
35S:C2	TOPO-C2-S	pGWB2	
35S:C3	TOPO-C3-S	pGWB2	
35S:C4	TOPO-C4-S	pGWB2	
35S:V2	TOPO-V2-S	pGWB2	
35S:CP	TOPO-CP-S	pGWB2	
35S:CP-GFP	TOPO-CP-NS	pGWB5	
35S:GFP-CP	TOPO-CP-S	pGWB6	
35S:RFP-CP	TOPO-CP-S	PGWB555	
35S:-CP-RFP	TOPO-CP-NS	PGWB554	
TYLCV infectious clone	TOPO-TYLCV	pGWB501	
CP-YFPn	TOPO-CP-NS	pGTQL1211YN	
CP-YFPc	TOPO-CP-NS	pGTQL1221YC	
