# Supplementary material for: Dynamic Virus-Dependent Subnuclear Localization of the Capsid Protein from a Geminivirus
Source: Front Plant Sci. 2017 Dec 22;8:2165. doi: 10.3389/fpls.2017.02165 (PMC5744400; doi:10.3389/fpls.2017.02165)
Supplement: TABLE S2 — Primers used for qPCR. [file Table_2.PDF]

Supplementary table 2: Primers used for qPCR

Primer Name	Sequence 5'-3'
qRep-F	TGAGAACGTCGTGTCTTCCG
qRep-R	TGACGTTGTACCACGCATCA
qC2-F	ACCTTCGTCACCCTCTACGA
qC2-R	AAACGCCATTCTCTGCCTGA
qC3-F	GGGGAACTCATCACTGCTCC
qC3-R	CTGAGGCTGTAATGTCGTCCA
qC4-F	ATCCGAACATTCAGGCAGCT
qC4-R	TGCTGACCTCCTCTAGCTGA
qV2-F	ATCTGTTGTAAGGGCCCGTG
qV2-R	CTTTCGGTACATGGGCCTGT
qCP-F	TGGAAGCAGCCCAATGGATT
qCP-R	GTTCTCGTACTTGGCTGCCT
ITS-F	ATAACCGCATCAGGTCTCCA
ITS-R	CCGAAGTTACGGATCCATTT
